# Genomic Insights into Host Susceptibility to Periprosthetic Joint Infections: A Comprehensive Literature Review

**DOI:** 10.3390/microorganisms12122486

**Published:** 2024-12-03

**Authors:** Juan D. Lizcano, Anabelle Visperas, Nicolas S. Piuzzi, Hesham Abdelbary, Carlos A. Higuera-Rueda

**Affiliations:** 1 Orthopedic Surgery Department, Cleveland Clinic, Weston, FL 33331, USA; lizcanj@ccf.org; 2 Orthopedic Surgery Department, Cleveland Clinic, Cleveland, OH 44195, USA; vispera@ccf.org (A.V.);; 3 Orthopedic Surgery Department, The Ottawa Hospital, Ontario, ON K1Y 4E9, Canada

**Keywords:** polymorphisms, PJI, arthroplasty, genes, septic revision

## Abstract

Periprosthetic joint infection (PJI) is a multifactorial disease, and the risk of contracting infection is determined by the complex interplays between environmental and host-related factors. While research has shown that certain individuals may have a genetic predisposition for PJI, the existing literature is scarce, and the heterogeneity in the assessed genes limits its clinical applicability. Our review on genetic susceptibility for PJI has the following two objectives: (1) Explore the potential risk of developing PJI based on specific genetic polymorphisms or allelic variations; and (2) Characterize the regulatory cascades involved in the risk of developing PJI. This review focused on clinical studies investigating the association between genetic mutations or variations with the development of PJI. The genes investigated in these studies included toll-like receptors and humoral pattern recognition molecules, cytokines, chemokines, mannose-binding lectin (MBL), bone metabolism molecules, and human leukocyte antigen. Among these genes, polymorphisms in IL-1, MBL, vitamin D receptors, HLA-C, and HLA-DQ might have a relevant impact on the development of PJI. The literature surrounding this topic is limited, but emerging transcriptomic and genome-wide association studies hold promise for identifying at-risk genes. This advancement could pave the way for incorporating genetic testing into preoperative risk stratification, enhancing personalized patient care.

## 1. Introduction

Periprosthetic joint infection (PJI) is a very complex and increasingly prevalent complication after total joint arthroplasty (TJA), making up one-third of all total knee revisions and almost one-quarter of all hip revisions in the United States [1,2]. Despite the novel treatment protocols and surgical alternatives, the incidence of PJI and failure rates after septic revision surgery remain constant throughout the years [3,4,5]. Due to the uptrend in PJI rates in recent years, new research surrounding host-related risk factors has been published [6].

The role of the intestinal microbiome [7], mental health [8,9], nutritional status [10,11], and medications [12] in the development of PJI are among the relevant topics. Conversely, genetic susceptibility to infection could be a determinant of all these factors but is often overlooked. Interestingly, the role of genes in joint arthroplasty has been thoroughly investigated for outcomes such as loosening and osteolysis [13,14,15,16] but not for PJI.

In 2017, Anderson et al. published a population-based cohort study to determine the presence of familiar clustering in PJI [17]. In this study, they found that first-degree relatives and combined first- and second-degree relatives had an increased risk of developing PJI. Additionally, they identified 116 high-risk pedigrees, 9 of which had a high ratio of observed PJI/TJA. Population-based studies allow for the identification of potential high-risk candidates for genotyping since the presence of high-risk pedigrees—in the absence of the relevant comorbidities—could indicate a heritable alteration in the DNA sequence, conferring a higher risk of getting this disease [17]. This heritable pattern affecting risk profile has been linked with single nucleotide polymorphisms (SNPs) in numerous immune regulatory cascades and specific human leukocyte antigen (HLA) genotypes [18,19,20]. SNP related to PJI incidence have been identified in protein receptors and intracellular mediators such as the osteoprotegerin system (RANK/RANKL/OPG), mannose-binding lectin (MBL), cytokines, chemokines, and toll-like receptors (TLR), as well as various proteins [21]. More recently, an association was found between HLA genotypes and the incidence of PJI [18]. However, the numerous genotypic variations published in the literature make it challenging for physicians to generate clinical recommendations. Being able to identify specific SNPs or immune genotypes linked to septic TJA failure would enable physicians to stratify the patients and generate appropriate perioperative recommendations based on modifiable risk factors to curb the risk of infection.

This manuscript aims to provide a comprehensive literature review of the current concepts of the genetic risk for PJI. Therefore, we will cover all relevant studies on this topic, emphasizing the specific genetic alterations and their repercussions. A summary of all SNP and genotypes evaluated in each study is included in Table 1. Similarly, this article will explore the specific mechanisms behind the identified genetic alterations and the future of genetic testing in PJI.

## 2. Toll Like Receptors and Humoral Pattern Recognition Molecules

**Figure 1:**
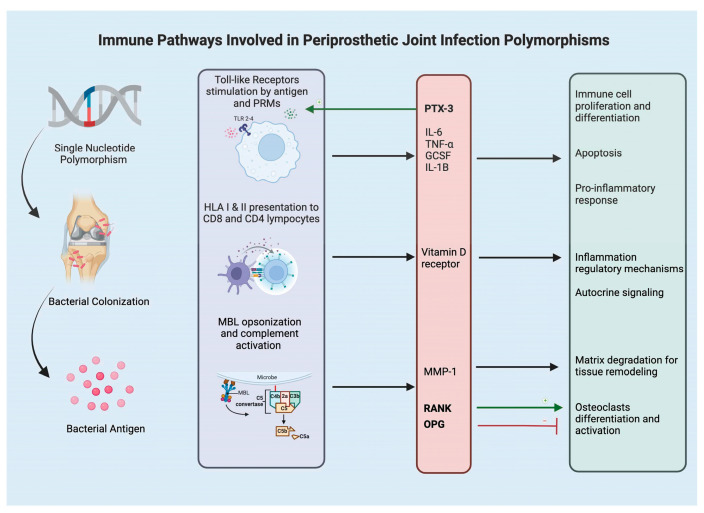
Diagram of immune pathways involved in periprosthetic joint infection polymorphisms.

Toll-like receptors (TLRs) and humoral pattern recognition molecules (PRMs) are key components of the innate immune system through the recognition of pathogen-associated molecular patterns (PAMPs) and damage-associated molecular patterns (DAMPs) [31]. These patterns are usually related to bacterial infections or released by damaged tissue. Across the broad family of TLRs, TLR-2 and TLR-4 have been identified as crucial components for initiating the response against Gram-positive and Gram-negative bacteria (Figure 1) [32,33,34]. A study by Galliera et al. found elevated serum TLR-2 levels in patients with chronic PJI [35]. Similarly, TLR-4 plays a role in Gram-negative lipopolysaccharide (LPS)-induced bone loss [36]. For these reasons, these receptors have been the target of studies investigating genetic alterations and the risk for PJI. El-Helou et al. found in an in vitro model that cells transfected with mutant TLR2 *R753Q* SNP showed an impaired response to the *Staphylococcus aureus* (*S. aureus*) peptidoglycan [22]. Interestingly, the prevalence of this SNP was not significantly different between patients with *S. aureus* PJI and non-infected controls. Similarly, Mrazek et al. studied the prevalence of missense polymorphisms in three genes coding for TLR-2 and TLR-4 in patients with and without PJI [23]. He found an equal distribution of these SNPs among the septic revisions, aseptic revisions, and healthy individuals.

Both studies investigated alterations in the same TLR-2 gene and, while the in vitro model showed an alteration in the TLR-2 function, the clinical models did not show any differences in this gene distribution. A possible explanation is the heterozygous status of infected patients for the altered gene, which leads to TLR-2 production in lower proportions, helping to maintain immune function [23]. Similarly, other mechanisms involving adaptative immune response and biofilm formation could have been involved in the PJI pathogenesis [37]. Considering that the etiologic agent in the study by Mrazek et al. was *Staphylococcus* Sp. in 60% of the cases, and that TLR-4 primarily responds to the lipopolysaccharide located in the wall of Gram-negative bacteria, the low prevalence of TLR-4 in PJI-positive patients is to be expected.

The long pentraxin 3 (PTX3) is a protein produced and secreted primarily by neutrophils that act as a PRM, facilitating pathogen recognition and phagocytosis by the immune cells [38]. This molecule was previously studied as a potential synovial marker for early PJI, showing excellent sensitivity and specificity for diagnosing PJI (AUC: 0.95) [39]. Granata et al. identified three common polymorphisms of PTX3 in patients with diagnosed PJI, aseptic complications, and healthy populations [19]. They found no differences in the prevalence of any SNPs between the infected and non-infected cases. Current data on the PJI risk of polymorphisms in the TLR and PRM molecules suggest that the mechanisms by which the immune system eradicates bacterial infections might not depend only on these innate immune system mediators. Another possible explanation worth noting is that the tested polymorphisms may be located in introns that do not code for proteins. Additionally, protein production could be influenced by post-translational modifications, which cannot be assessed at the genetic level.

## 3. Cytokines and Chemokines

Cytokines are the primary signaling proteins of the immune system. Interleukins (ILs) have been the most studied immune system proteins in relation to PJI. IL-1B is a proinflammatory cytokine that has been found in association with Staphylococcal PJI in synovial fluid and blood [40,41]. IL-1B has been proposed as a marker that could aid in differentiating *S. aureus* from other pathogens, such as *Staphylococcus epidermidis (S. epidermidis)*, in septic total joint revisions [42]. Three studies have investigated the repercussions of IL-1B genetic polymorphisms in the incidence of PJI. Stahelova et al. performed a case-control study to explore the incidence of SNP in high-risk cytokines for PJI. Among all six SNPs tested, the *IL-1B-511* polymorphism was the only one expressing higher carriage rates in the infected patients compared to the aseptic revisions (69% vs. 51%, *p* = 0.006) and the healthy controls (69% vs. 55%, *p* = 0.04) [24]. Similarly, Granata et al. evaluated eight SNPs in four different pro-inflammatory cytokines and found that only the IL-1B *rs2853550* polymorphism was associated with a higher probability of infection (OR = 4.05; 95%CI = 1.28–12.93) [19]. In a case-control study by Erdemli et al., patients with and without PJI were screened for specific SNPs. Among the tested genes was the IL-1 ribonuclease 1 variable number tandem repeat (*RN-VNTR*) polymorphism, which codes for a promoter region of the *IL-1B* gene. They found that two *IL-1RN* SNP-specific haplotypes increased the risk of developing septic failure (*p* = 0.002) [25]. These studies imply a causal relationship between PJI and the IL-1 inflammatory pathways.

In the same study by Erdemli et al., granulocyte colony-stimulating factors (GCSFs) and IL-6 SNPs were also analyzed [25]. In the case of *GCSF3R*, the gene encodes for the GCSF receptor (GCSFR), which plays an important role in the proliferation and differentiation of myeloid progenitor cells into neutrophils [43]. For the *GCSF3R* SNP, one specific allele was independently associated with PJI risk (OR: 9.31; *p* = 0.002). Moreover, the IL-6-174 gene polymorphism was also described as an independent risk factor for failure. IL-6 is an important cytokine involved in the innate and adaptative immune system response, especially against bacteria and fungi [44]. Several other studies in the literature analyzing the impact of SNP on the IL-6 gene yielded contrasting results. Two case-control cohorts analyzing the *IL-6-174* SNPs on PJI and aseptic patients found no differences in incidence [24,26]. Moreover, in the article by Granata et al., the *IL-6-597* and *IL-6-572* SNPs were found to be in similar proportion between the infected and non-infected patients. The role of the IL-6 gene polymorphisms in predisposing to PJI is still a matter of debate [19].

Tumor necrosis factor-alpha (TNF- α) is a crucial proinflammatory protein primarily produced by macrophages to amplify the immune response, induce inflammatory gene expression, and promote cell death [45]. TNF-α upregulates alpha-defensin production, which is currently a widely used marker for diagnosing PJI [46]. Likewise, some studies suggest that using TNF-α blockers is linked with increased PJI rates; however, there are conflicting results in various systematic reviews [47]. Studies measuring the TNF-α gene polymorphism contribution to PJI reported a similar trend. Erdemli et al. found a higher frequency of *TNF-α-238* SNPs among patients with PJI compared to patients undergoing aseptic revisions [25]. Contrastingly, Stahelova et al. performed a similar study, measuring the *TNF-α-308* and *TNF-α-238* SNPs in healthy individuals, as well as septic and aseptic revisions, with no differences in SNP distributions noted across the groups [24].

Immune markers characteristic of T-helper 17 (Th-17) lymphocytes’ innate response to infection have been found to be elevated in the synovial fluid of patients with PJI [41,48]. Navratilova et al. investigated the effect of polymorphisms in cytokines and chemokines commonly related to the Th-17 immune response in relation to PJI. Their results suggest that the SNPs of IL-17A, IL-17F, IL-4, IL-12A, IL-12B, IL-23R, chemokine ligand (CXCL) 1, CXCL5, and CXCR2 are not found in a higher frequency in patients with PJI compared to aseptic revisions [49].

## 4. Mannose-Binding Lectin

Soluble C-type lectin receptors are a type of pattern recognition receptor that includes a liver-based mannose-binding lectin (MBL). The MBL binds to the mannose-rich component of the PAMPs, serving as an opsonin, initiating complement activation, and facilitating phagocytosis. The lectin pathway of the complement represents one of the main mechanisms of the innate immune system to clear out infection [50]. A high rate of MBL haplotype variation has been documented across different populations, with associated variations in MBL levels, which could increase susceptibility to infection [51]. Malik et al. analyzed MBL gene mutations in chromosome 10 associated with codons 52 and 54, as well as the promoter in positions *−550* and *−221*. A specific allelic and genotype frequency within the SNP *550* and codon *54* were found to be more prevalent in the septic group compared to healthy individuals but with a similar distribution compared to aseptic revisions [27]. In a similar study, Navratilova et al. measured MBL −*550*, −*221*, and *+54* SNPs on patients with PJI, aseptic revisions, and healthy controls [28]. He found that an MBL, *−550* SNP, was not only more frequent in infected cases but also that carriers of this polymorphism had lower serum MBL levels (median; 593 vs. 1876 ng/mL; *p* < 0.01). The latter study questioned whether the mutation could lead to different clinical manifestations in terms of MBL production and independent risk profiles for developing infection.

## 5. Bone Metabolism (VDR, OPG, MMP)

The osteoprotegerin (OPG) and its interaction with the receptor activator of nuclear factor kappa B ligand (RANK) and its ligand (RANKL) are relevant molecules in the mechanisms of infection-related bone loss and osteolysis. An animal study showed decreased bone resorption in bone infected with *S. aureus* as a consequence of RANKL inhibition and decreased osteoclast formation [52]. The role of this system in the pathogenesis of infection is less clear, and allelic variations in these genes have only been associated with decreased bone mineral density [53]. Malik et al. sought to investigate the prevalence of four different SNPs in the OPG *(−153, −245, +1181*) and RANK *(+575*) genes. After submitting an addendum for their results, the gene frequencies were comparable between the healthy controls, septic, and aseptic revisions [29]. Similarly, Navratilova et al. measured the incidence of *OPG-163* SNP in patients with PJI and compared it to non-infected controls, finding no difference between the groups [30]. Another crucial group of proteins involved in bone remodeling and osteolytic processes after TJA is the matrix metalloproteinases (MMPs). MMP expression is upregulated by inflammatory markers such as LPS, TNF-alpha, and IL-1 [54]. These proteins were found in high concentrations in the implant–bone interface of loose prostheses [55]. Genetic studies found that an SNP in a promoter region of the MMP-1 gene and a *−1607 1G/2G* SNP of this same gene might contribute to the development of aseptic loosening and osteomyelitis, respectively [56,57]. MMP-1 genetic influence in PJI has only been investigated by Malik et al. in a retrospective case-control study. However, none of the four different MMP-1 polymorphisms tested were found in a higher frequency in patients with PJI [26]. The *OPG-163* and *MMP-1* gene SNPs seem to have a more relevant role in the development of mechanical complications and osteoporosis pathogenesis rather than increasing PJI predisposition [58,59].

Furthermore, the vitamin D receptor (VDR) plays a central role in calcium metabolism, including immune modulation and regulation of cell growth and differentiation [60]. For this reason, the VDR gene has been described to have a “pleiotropic” role, and its polymorphism is associated with a wide array of autoimmune disorders and protection against infection [61,62,63,64,65,66]. The prevalence of *VDR-T* and *VDR-L* SNPs in patients who underwent septic revision TJA surgery was investigated by Malik et al. They found that the T allele (OR = 1.76; 95% CI 1.16 to 2.66, *p* = 0.007) and T/T genotype (*p* = 0.028) for *VDR-T* were significantly associated with osteolysis in the setting of PJI [26]. Infections related to SNPs in VDR have been described in other fields of medicine, which could be related to the presence of this receptor in lymphocytes and antigen-presenting cells for autocrine signaling through the production of active vitamin D metabolite [67,68,69]. VDR also contributes to intestinal homeostasis and bacterial invasion by downregulating bacterial-stimulated NF-κB activity in the intestine [70]. As only recently discovered, bacterial intestinal translocation and gut microbiome play an essential role in PJI pathogenesis [7,71]. Future genetic profiling studies should focus on the association between PJI and VDR.

## 6. Human Leukocyte Antigen

The human leukocyte antigen (HLA) is the name given to the human major histocompatibility complex (MHC), which is a group of glycoproteins responsible for recognizing endogenous from exogenous antigens. The HLA is a central component of the innate and adaptative immune response. Its genomic sequence on chromosome 6 encodes for the following three different types of proteins: Class I (HLA-A, B, C, E, F, G, H) which are responsible for defense against intracellular pathogens; Class II (HLA-DP, DQ, DR, DM) which are in the surface of antigen-presenting cells and responsible for defense against extracellular pathogens; and Class III which are components of the complement system, 21-hydroxylase, heat shock protein, and tumor necrosis factors [72,73]. Due to the major influence of HLA in initiating the immune response to pathogens, multiple studies have identified at-risk mutations for specific conditions such as tuberculosis, leprosy, melioidosis, and staphylococcal infections [72]. The *S. aureus* superantigen involved in toxic shock syndrome was found to bind to the HLA-DR1 molecule, and SNPs in this region were established to be determinants in the antibody production against this toxin [74]. Additionally, two case-control studies showed that SNPs in the HLA class II region, more specifically in the *HLA-DRA* and *DRB1* genes, increased susceptibility to *S. aureus* infections [75,76].

Recently, Neufeld et al. published a novel study analyzing the relationship between HLA gene polymorphisms and the risk of developing PJI using a matched cohort of infected and non-infected TJA revisions. They analyzed 11 different HLA loci coding for class I and II proteins (HLA-A, -B, and -C; HLA-DRB1, -DRB3/4/5, -DQA1, -DQB1, -DPA1, and -DPB1). They found increased risk of PJI in three different alleles, including *HLA-C∗06:02* (OR 5.25, 95% CI 0.96 to 28.6, *p* = 0.064), *HLA-DQA1∗04:01* (*p* = 0.096), and *HLA-DQB1∗04:02* (*p* = 0.096). The *HLA-C∗03:04* (OR 0.12, 95% CI 0.01 to 1.10, *p* = 0.052) was found to be a protective factor for PJI [18]. These results contrast with the previous literature and introduce Class I HLA-C genes as determinants for *S. aureus* infection risk [66,67]. The complex interplays among chronic prosthetic infection, biofilm formation, and HLA heterogeneity in this patient population may contribute to the unique HLA response to infection in PJI.

## 7. Future of Genetic Testing and PJI

The literature surrounding this topic is limited, but emerging transcriptomic and genome-wide association studies hold promise for identifying at-risk genes. This advancement could pave the way for incorporating genetic testing into preoperative risk stratification, enhancing personalized patient care. New diagnostic tools, such as transcriptomics testing on synovial fluid, have been efficiently used to recognize upregulated gene expression of immune-related molecules and could potentially be used in the diagnosis of PJI. Masters et al. utilized transcriptomics and found 28 PJI-associated genes that had increased expression in synovial fluid [42]. Moreover, in the same study, IL-13RA2, IL-17D, and MMP3 were higher in the *S. aureus* and *S. epidermidis* compared to other microorganisms. The role of IL-13RA2 and IL-17D in bacterial pathogenesis has not been well delineated, but the higher levels of these cytokines in staphylococcal infections suggest that the immune mediators are dependent upon the type of infecting microorganism [77,78]. This poses a bigger challenge in the integration of genetic testing into the field of orthopedic infection prevention. While this technology is primarily used to identify pathogens and potential diagnostic markers, its utility as a tool for gene identification should not be overlooked, as these could be used as targets in future genetic risk profiling studies.

Another effective strategy is genome-wide association studies, which identify specific gene variants or traits in large populations to uncover the genetic factors contributing to complex diseases such as PJI. Guo et al. performed a genome-wide association study comparing patients who had a TJA-related mechanical complication and PJI to a control group. They found that an SNP in a locus near the *RBM26* gene reached genome-wide significance for PJI [79]. Similarly, in a recent genome-wide association study, Chen et al. was able to identify four immune-related (*PLCB1*) and non-immune-related (*SAMD4B*, *STAG1*, *EXD3*) gene SNPs to obtain the polygenic risk scores for developing surgical site infection after TKA [80]. While the clinical value of these results is uncertain, identifying at-risk genes at a population level could improve the applicability of genetic testing strategies for infection prevention. Furthermore, the recent discovery of the HLA subtype as a risk factor for PJI represents a big advancement in the study of genomics and infection risk. Characterizing an individual’s overall immune profile rather than a specific immune mediator allows for a broader genetic perspective and provides insights into population-level susceptibility to PJI at a cellular level [6].

## 8. Conclusions

There is a lack of sufficient high-quality studies on the genetics of PJI, which limits the integration of genetic strategies into clinical practice. However, existing research suggests that SNPs in genes such as IL-1, MBL, vitamin D receptors, HLA-C, and HLA-DQ may play a significant role in the development of PJI. The slow progress in this field over the past decade underscores the need for researchers to adopt advanced tools, such as transcriptomics and genome-wide association studies to better identify host-specific risk factors for PJI. As prevention remains the most effective strategy against this increasingly common complication, more genetic and host risk profiling studies should be conducted in patients undergoing a primary total joint arthroplasty (TJA).

## Figures and Tables

**Table 1:** Studies’ characteristics, polymorphisms, and genotypes.

Risk Factor	Author	Reference Number	SNP/Genotype	Alleles	Number of Subjects Tested for SNP		
					PJI Group	Aseptic Control Group	Healthy Control Group
TLR-2	El-Helou (2011)	[22]	rs5743708	G/A	66	-	57
	Mrazek (2013)	[23]	rs5743708	G/A	98	-	252
TL-4	Mrazek (2013)	[23]	rs4986790	A/G,T	98	-	252
			rs4986791	C/T	98	-	252
PTX3	Granata ^§^ (2014)	[19]	rs2305619	A/G,T	46	-	47
			rs3816527	C/A,T	46	-	47
			rs1840680	A/C,G,T	46	-	47
IL-1B	Stahelova (2012)	[24]	rs16944	A/G	89	214	188
			rs1143634	G/A	89	214	188
	Granata (2014)	[19]	rs2853550	A/G,T	46	-	47
	Erdemli (2018)	[25]	rs1143623	C/A,G	36	52	-
GCSF	Erdemli (2018)	[25]	rs3769817	T/A,C,G	36	52	-
IL-6	Erdemli (2018)	[25]	rs1800795	C/G,T	36	52	-
	Malik (2007)	[26]	rs1800795	C/G,T	63	88	188
	Stahelova (2012)	[24]	rs1800795	C/G,T	89	214	188
		[24]	rs1800796	G/A,C	89	214	188
	Granata (2024)	[19]	rs1800796	G/A,C	46	-	47
		[19]	rs1800797	A/C,G,T	46	-	47
TNF-α	Erdemli (2018)	[25]	rs361525	G/A	36	52	-
	Stahelova (2012)	[24]	rs361525	G/A	89	214	188
			rs1800629	G/A	89	214	188
MLB	Malik (2007)	[27]	rs11003125	G/C	144	91	62
			rs7096206	G/A,C,T	148	91	62
			rs5030737	G/A,T	145	91	62
			rs1800450	C/T	148	91	61
	Navratilova (2012)	[28]	rs11003125	G/C	112	245	196
			rs7096206	G/A,C,T	112	245	196
			rs1800450	G/A	112	245	196
OPG	Malik (2006-2009)	[29]	rs2073618	G/C	53	91	150
			rs2073617	G/A,T	62	91	149
			rs3102735	A/C,G,T	62	89	147
	Navratilova (2012)	[30]	rs3102735	A/C,G,T	185	251	98
RANK	Malik (2006-2009)	[29]	rs1805034	C/T	62	85	144
MMP-1	Malik ^‡^ (2007)	[26]	rs5854	G/A	62	87	148
			rs2397776	T/C	62	88	148
			rs470747	A/G	62	89	147
VDR	Malik ^‡^ (2007)	[26]	rs731236	A/G,T	63	88	148
HLA	Neufeld (2024)	[18]	-	HLA-C∗06:02	23	-	26
			-	HLA-DQA1∗04:01	23	-	26
			-	HLA-DQB1∗04:02	23	-	26
			-	HLA-C∗03:04	23	-	26

^§^ Three most common PTX3 SNP of 14 tested in the study; ^‡^ MMP-1-2 and VDR-L not depicted due to monomorphic distribution; * TH-17 related cytokines (IL-17A, IL-17F, IL-4, IL-12A, IL-12B, IL-23R, CXCL1, CXCL5, and CXCR2) were excluded from the table.

## Data Availability

No new data were created or analyzed in this study. Data sharing is not applicable to this article.

## Abbreviations

PJI: periprosthetic joint infection; TJA, total joint arthroplasty; SNP, single nucleotide polymorphism; HLA, human leukocyte antigen; RANK, receptor activator of nuclear factor kappa-B; RANKL, receptor activator of nuclear factor kappa-B ligand; OPG, osteoprotegerin; MBL, mannose-binding lectin; TLR, toll-like receptor; PRMs, pattern recognition molecules; PAMPs, pathogen-associated molecular patterns; DAMPs, damage-associated molecular patterns; PTX3, pentraxin 3; IL, interleukin; G-CSF, granulocyte colony-stimulating factor; TNF-α, tumor necrosis factor-alpha; CXCL, C-X-C motif chemokine ligand; VDR, vitamin D receptor; MHC, major histocompatibility complex.

## References

[1] (2023). American Joint Replacement Registry (AJRR) 2023 Anual Report.

[2] Siddiqi A., Warren J.A., Manrique-Succar J., Molloy R.M., Barsoum W.K., Piuzzi N.S. (2021). Temporal Trends in Revision Total Hip and Knee Arthroplasty from 2008 to 2018: Gaps and Opportunities. J. Bone Jt. Surg..

[3] Corona P.S., Vicente M., Carrera L., Rodríguez-Pardo D., Corró S. (2020). Current Actual Success Rate of the Two-Stage Exchange Arthroplasty Strategy in Chronic Hip and Knee Periprosthetic Joint Infection: Insights into Non-Completed Second-Stage Cases. Bone Jt. J..

[4] Barros L.H., Barbosa T.A., Esteves J., Abreu M., Soares D., Sousa R. (2019). Early Debridement, Antibiotics and Implant Retention (DAIR) in Patients with Suspected Acute Infection after Hip or Knee Arthroplasty—Safe, Effective and without Negative Functional Impact. J. Bone Jt. Infect..

[5] Jevnikar B.E., Khan S.T., Huffman N., Pasqualini I., Surace P.A., Deren M.E., Piuzzi N.S. (2024). Advancements in Treatment Strategies for Periprosthetic Joint Infections: A Comprehensive Review. J. Clin. Orthop. Trauma.

[6] Piuzzi N.S., Klika A.K., Lu Q., Higuera-Rueda C.A., Stappenbeck T., Visperas A. (2024). Periprosthetic Joint Infection and Immunity: Current Understanding of Host–Microbe Interplay. J. Orthop. Res..

[7] Chisari E., Cho J., Wouthuyzen-Bakker M., Parvizi J. (2022). Periprosthetic Joint Infection and the Trojan Horse Theory: Examining the Role of Gut Dysbiosis and Epithelial Integrity. J. Arthroplast..

[8] Harmer J.R., Wyles C.C., Duong S.Q., Morgan Iii R.J., Maradit-Kremers H., Abdel M.P. (2023). Depression and Anxiety Are Associated with an Increased Risk of Infection, Revision, and Reoperation Following Total Hip or Knee Arthroplasty. Bone Jt. J..

[9] Anis H.K., Warren J.A., Klika A.K., Navale S.M., Zhou G., Barsoum W.K., Higuera C.A., Piuzzi N.S. (2022). Greater Prevalence of Mental Health Conditions in Septic Revision Total Knee Arthroplasty: A Call to Action. J. Knee Surg..

[10] Scarcella N.R., Mills F.B., Seidelman J.L., Jiranek W.A. (2024). The Effect of Nutritional Status in the Treatment of Periprosthetic Joint Infections in Total Hip Arthroplasty. J. Arthroplast..

[11] Emara A.K., Hadad M.J., Dube M., Klika A.K., Burguera B., Piuzzi N.S. (2022). Team Approach: Nutritional Assessment and Interventions in Elective Hip and Knee Arthroplasty. JBJS Rev..

[12] Magruder M.L., Yao V.J.H., Rodriguez A.N., Ng M.K., Sasson V., Erez O. (2023). Does Semaglutide Use Decrease Complications and Costs Following Total Knee Arthroplasty?. J. Arthroplast..

[13] Koks S., Wood D.J., Reimann E., Awiszus F., Lohmann C.H., Bertrand J., Prans E., Maasalu K., Märtson A. (2020). The Genetic Variations Associated with Time to Aseptic Loosening After Total Joint Arthroplasty. J. Arthroplast..

[14] Veronesi F., Tschon M., Fini M. (2017). Gene Expression in Osteolysis: Review on the Identification of Altered Molecular Pathways in Preclinical and Clinical Studies. Int. J. Mol. Sci..

[15] MacInnes S.J., Hatzikotoulas K., Fenstad A.M., Shah K., Southam L., Tachmazidou I., Hallan G., Dale H., Panoutsopoulou K., Furnes O. (2019). The 2018 Otto Aufranc Award: How Does Genome-Wide Variation Affect Osteolysis Risk After THA?. Clin. Orthop. Relat. Res..

[16] Brüggemann A., Eriksson N., Michaëlsson K., Hailer N.P. (2022). Risk of Revision After Arthroplasty Associated with Specific Gene Loci: A Genomewide Association Study of Single-Nucleotide Polymorphisms in 1,130 Twins Treated with Arthroplasty. J. Bone Jt. Surg..

[17] Anderson M.B., Curtin K., Wong J., Pelt C.E., Peters C.L., Gililland J.M. (2017). Familial Clustering Identified in Periprosthetic Joint Infection Following Primary Total Joint Arthroplasty: A Population-Based Cohort Study. J. Bone Jt. Surg..

[18] Neufeld M.E., Sheridan G.A., MacDonell T., Howard L.C., Masri B.A., Keown P., Sherwood K., Garbuz D.S. (2024). The John Charnley Award: The Impact of Human Leukocyte Antigen Genotype on Bacterial Infection Rates and Successful Eradication in Total Hip Arthroplasty. J. Arthroplast..

[19] Granata V., Strina D., Possetti V., Leone R., Valentino S., Chiappetta K., Loppini M., Mantovani A., Bottazzi B., Asselta R. (2024). Interleukin-1β Polymorphisms Are Genetic Markers of Susceptibility to Periprosthetic Joint Infection in Total Hip and Knee Arthroplasty. Genes.

[20] Hijazi A., Hasan A., Pearl A., Memon R., Debeau M., Roldan M., Awad M.E., Abdul-Kabir E., Saleh K.J. (2022). Genetic Polymorphisms Associated with Perioperative Joint Infection Following Total Joint Arthroplasty: A Systematic Review and Meta-Analysis. Antibiotics.

[21] Zhou X., Yishake M., Li J., Jiang L., Wu L., Liu R., Xu N. (2015). Genetic Susceptibility to Prosthetic Joint Infection Following Total Joint Arthroplasty: A Systematic Review. Gene.

[22] El-Helou O., Berbari E.F., Brown R.A., Gralewski J.H., Osmon D.R., Razonable R.R. (2011). Functional Assessment of Toll-like Receptor 2 and Its Relevance in Patients with Staphylococcus Aureus Infection of Joint Prosthesis. Hum. Immunol..

[23] Mrazek F., Gallo J., Stahelova A., Petrek M. (2013). Coding Variants of TLR2 and TLR4 Genes Do Not Substantially Contribute to Prosthetic Joint Infection. Inflamm. Res..

[24] Stahelova A., Mrazek F., Smizansky M., Petrek M., Gallo J. (2012). Variation in the IL1B, TNF and IL6 Genes and Individual Susceptibility to Prosthetic Joint Infection. BMC Immunol.

[25] Erdemli B., Özbek E.A., Başarir K., Karahan Z.C., Öcal D., Biriken D. (2018). Proinflammatory Biomarkers’ Level and Functional Genetic Polymorphisms in Periprosthetic Joint Infection. Acta Orthop. Traumatol. Turc..

[26] Malik M.H.A., Jury F., Bayat A., Ollier W.E.R., Kay P.R. (2007). Genetic Susceptibility to Total Hip Arthroplasty Failure: A Preliminary Study on the Influence of Matrix Metalloproteinase 1, Interleukin 6 Polymorphisms and Vitamin D Receptor. Ann. Rheum Dis..

[27] Malik M.H.A., Bayat A., Jury F., Kay P.R., Ollier W.E.R. (2007). Genetic Susceptibility to Total Hip Arthroplasty Failure—Positive Association with Mannose-Binding Lectin. J. Arthroplast..

[28] Navratilova Z., Gallo J., Mrazek F., Lostak J., Petrek M. (2012). MBL2 Gene Variation Affecting Serum MBL Is Associated with Prosthetic Joint Infection in Czech Patients after Total Joint Arthroplasty. Tissue Antigens.

[29] Malik M.H.A., Bayat A., Jury F., Ollier W.E.R., Kay P.R. (2006). Genetic Susceptibility to Hip Arthroplasty Failure—Association with the RANK/OPG Pathway. Int. Orthop. (SICO).

[30] Navratilova Z., Gallo J., Smizansky M., Mrazek F., Petrek M. (2014). Osteoprotegerin Gene Polymorphism Is Not Associated with Prosthetic Joint Infection after Total Joint Arthroplasty in the Czech Population. Biomed. Pap. Med. Fac. Univ. Palacky Olomouc. Czech. Repub..

[31] Molteni M., Gemma S., Rossetti C. (2016). The Role of Toll-Like Receptor 4 in Infectious and Noninfectious Inflammation. Mediat. Inflamm..

[32] Simpson M.E., Petri W.A. (2020). TLR2 as a Therapeutic Target in Bacterial Infection. Trends Mol. Med..

[33] Hanzelmann D., Joo H.-S., Franz-Wachtel M., Hertlein T., Stevanovic S., Macek B., Wolz C., Götz F., Otto M., Kretschmer D. (2016). Toll-like Receptor 2 Activation Depends on Lipopeptide Shedding by Bacterial Surfactants. Nat. Commun..

[34] Elson G., Dunn-Siegrist I., Daubeuf B., Pugin J. (2007). Contribution of Toll-like Receptors to the Innate Immune Response to Gram-Negative and Gram-Positive Bacteria. Blood.

[35] Galliera E., Drago L., Vassena C., Romanò C., Gioia Marazzi M., Salcito L., Corsi Romanelli M.M. (2014). Toll-Like Receptor 2 in Serum: A Potential Diagnostic Marker of Prosthetic Joint Infection?. J. Clin. Microbiol..

[36] Chen M.-F., Chang C.-H., Hu C.-C., Wu Y.-Y., Chang Y., Ueng S.W.N. (2019). Periprosthetic Joint Infection Caused by Gram-Positive Versus Gram-Negative Bacteria: Lipopolysaccharide, but Not Lipoteichoic Acid, Exerts Adverse Osteoclast-Mediated Effects on the Bone. J. Clin. Med..

[37] Visperas A., Santana D., Klika A.K., Higuera-Rueda C.A., Piuzzi N.S. (2022). Current Treatments for Biofilm-associated Periprosthetic Joint Infection and New Potential Strategies. J. Orthop. Res..

[38] Porte R., Davoudian S., Asgari F., Parente R., Mantovani A., Garlanda C., Bottazzi B. (2019). The Long Pentraxin PTX3 as a Humoral Innate Immunity Functional Player and Biomarker of Infections and Sepsis. Front. Immunol..

[39] Loppini M., Di Maio M., Avigni R., Leone R., Inforzato A., Grappiolo G., Mantovani A., Bottazzi B. (2023). Long Pentraxin 3 as a New Biomarker for Diagnosis of Hip and Knee Periprosthetic Joint Infections. J. Clin. Med..

[40] Gollwitzer H., Dombrowski Y., Prodinger P.M., Peric M., Summer B., Hapfelmeier A., Saldamli B., Pankow F., Von Eisenhart-Rothe R., Imhoff A.B. (2013). Antimicrobial Peptides and Proinflammatory Cytokines in Periprosthetic Joint Infection. J. Bone Jt. Surg..

[41] Deirmengian C., Hallab N., Tarabishy A., Valle C.D., Jacobs J.J., Lonner J., Booth R.E. (2010). Synovial Fluid Biomarkers for Periprosthetic Infection. Clin. Orthop. Relat. Res..

[42] Masters T.L., Bhagwate A.V., Dehankar M.K., Greenwood-Quaintance K.E., Abdel M.P., Mandrekar J.N., Patel R. (2022). Human Transcriptomic Response to Periprosthetic Joint Infection. Gene.

[43] Dwivedi P., Greis K.D. (2017). Granulocyte Colony Stimulating Factor Receptor (G-CSFR) Signaling in Severe Congenital Neutropenia, Chronic Neutrophilic Leukemia and Related Malignancies. Exp. Hematol..

[44] Rose-John S., Winthrop K., Calabrese L. (2017). The Role of IL-6 in Host Defence against Infections: Immunobiology and Clinical Implications. Nat. Rev. Rheumatol..

[45] Van Loo G., Bertrand M.J.M. (2023). Death by TNF: A Road to Inflammation. Nat. Rev. Immunol..

[46] Arvieux C., Common H. (2019). New Diagnostic Tools for Prosthetic Joint Infection. Orthop. Traumatol. Surg. Res..

[47] Yeganeh M.H., Kheir M.M., Shahi A., Parvizi J. (2018). Rheumatoid Arthritis, Disease Modifying Agents, and Periprosthetic Joint Infection: What Does a Joint Surgeon Need to Know?. J. Arthroplast..

[48] Qin L., Du C., Yang J., Wang H., Su X., Wei L., Zhao C., Chen C., Chen H., Hu N. (2022). Synovial Fluid Interleukin Levels Cannot Distinguish between Prosthetic Joint Infection and Active Rheumatoid Arthritis after Hip or Knee Arthroplasty. Diagnostics.

[49] Navratilova Z., Gallo J., Mrazek F., Petrek M. (2012). Genetic Variation in Key Molecules of the Th-17 Immune Response Is Not Associated with Risk for Prosthetic Joint Infection in a Czech Population. Biomed. Pap. Med. Fac. Univ. Palacky Olomouc. Czech. Repub..

[50] Kalia N., Singh J., Kaur M. (2021). The Ambiguous Role of Mannose-Binding Lectin (MBL) in Human Immunity. Open Med..

[51] Takahashi K., Ezekowitz R.A.B. (2005). The Role of the Mannose-Binding Lectin in Innate Immunity. Clin. Infect. Dis..

[52] Campbell M.J., Bustamante-Gomez C., Fu Q., Beenken K.E., Reyes-Pardo H., Smeltzer M.S., O’Brien C.A. (2024). RANKL-Mediated Osteoclast Formation Is Required for Bone Loss in a Murine Model of Staphylococcus Aureus Osteomyelitis. Bone.

[53] Takács I., Lazáry Á., Kósa J.P., Kiss J., Balla B., Nagy Z., Bácsi K., Speer G., Lakatos P. (2010). Allelic Variations of RANKL/OPG Signaling System Are Related to Bone Mineral Density and in Vivo Gene Expression. Eur. J. Endocrinol..

[54] Fujisaki K., Tanabe N., Suzuki N., Mitsui N., Oka H., Ito K., Maeno M. (2006). The Effect of IL-1α on the Expression of Matrix Metalloproteinases, Plasminogen Activators, and Their Inhibitors in Osteoblastic ROS 17/2.8 Cells. Life Sci..

[55] Takagi M., Konttinen Y.T., Kemppinen P., Sorsa T., Tschesche H., Bläser J., Suda A., Santavirta S. (1995). Tissue Inhibitor of Metalloproteinase 1, Collagenolytic and Gelatinolytic Activity in Loose Hip Endoprostheses. J. Rheumatol..

[56] Godoy-Santos A.L., D’Elia C.O., Teixeira W.J., Cabrita H.B., Camanho G.L. (2009). Aseptic Loosening of Total Hip Arthroplasty: Preliminary Genetic Investigation. J. Arthroplast..

[57] Montes A.H., Valle-Garay E., Alvarez V., Pevida M., García Pérez E., Paz J., Meana A., Asensi V. (2010). A Functional Polymorphism in *MMP1* Could Influence Osteomyelitis Development. J. Bone Miner. Res..

[58] Ding J., Zhang C., Guo Y. (2021). The Association of OPG Polymorphisms with Risk of Osteoporotic Fractures: A Systematic Review and Meta-Analysis. Medicine.

[59] Guo L., Tang K., Quan Z., Zhao Z., Jiang D. (2014). Association Between Seven Common *OPG* Genetic Polymorphisms and Osteoporosis Risk: A Meta-Analysis. DNA Cell Biol..

[60] Pasqualini I., Huffman N., Keller S.F., McLaughlin J.P., Molloy R.M., Deren M.E., Piuzzi N.S. (2023). Team Approach: Bone Health Optimization in Orthopaedic Surgery. JBJS Rev..

[61] Uitterlinden A.G., Fang Y., Van Meurs J.B.J., Van Leeuwen H., Pols H.A.P. (2004). Vitamin D Receptor Gene Polymorphisms in Relation to Vitamin D Related Disease States. J. Steroid Biochem. Mol. Biol..

[62] Mohammadifard N., Sadeghian L., Hassannejad R., Khosravi E., Gharipour M., Karimi S., Hosseini S., Sepahifar M., Bahrami G., Haghighatdoost F. (2024). Comparing Vitamin D Receptor Gene Polymorphisms in Rs11568820, Rs7970314, Rs4334089 between COVID-19 Patients with Mild and Severe Symptoms: A Case Control Study. Sci. Rep..

[63] Zhai N., Bidares R., Makoui M.H., Aslani S., Mohammadi P., Razi B., Imani D., Yazdchi M., Mikaeili H. (2020). Vitamin D Receptor Gene Polymorphisms and the Risk of the Type 1 Diabetes: A Meta-Regression and Updated Meta-Analysis. BMC Endocr. Disord..

[64] Smolders J., Peelen E., Thewissen M., Menheere P., Cohen Tervaert J.W., Hupperts R., Damoiseaux J. (2009). The Relevance of Vitamin D Receptor Gene Polymorphisms for Vitamin D Research in Multiple Sclerosis. Autoimmun. Rev..

[65] Emara A.K., Nageeb E., George J., Buttaro M.A., Higuera C., Piuzzi N.S. (2020). Hypovitaminosis D in Lower Extremity Joint Arthroplasty: A Systematic Review and Meta-Analysis. J. Orthop..

[66] Piuzzi N.S., George J., Khlopas A., Klika A.K., Mont M.A., Muschler G.F., Higuera C.A. (2018). High Prevalence and Seasonal Variation of Hypovitaminosis D in Patients Scheduled for Lower Extremity Total Joint Arthroplasty. Ann. Transl. Med..

[67] Aranow C. (2011). Vitamin D and the Immune System. J. Investig. Med..

[68] Zacharioudaki M., Messaritakis I., Galanakis E. (2021). Vitamin D Receptor, Vitamin D Binding Protein and CYP27B1 Single Nucleotide Polymorphisms and Susceptibility to Viral Infections in Infants. Sci. Rep..

[69] Roth D.E., Jones A.B., Prosser C., Robinson J.L., Vohra S. (2008). Vitamin D Receptor Polymorphisms and the Risk of Acute Lower Respiratory Tract Infection in Early Childhood. J. Infect. Dis..

[70] Wu S., Liao A.P., Xia Y., Chun Li Y., Li J.-D., Sartor R.B., Sun J. (2010). Vitamin D Receptor Negatively Regulates Bacterial-Stimulated NF-κB Activity in Intestine. Am. J. Pathol..

[71] Chisari E., D’Mello D., Sherman M.B., Parvizi J. (2022). Inflammatory Bowel Diseases Increase the Risk of Periprosthetic Joint Infection. J. Bone Jt. Surg..

[72] Medhasi S., Chantratita N. (2022). Human Leukocyte Antigen (HLA) System: Genetics and Association with Bacterial and Viral Infections. J. Immunol. Res..

[73] Crux N.B., Elahi S. (2017). Human Leukocyte Antigen (HLA) and Immune Regulation: How Do Classical and Non-Classical HLA Alleles Modulate Immune Response to Human Immunodeficiency Virus and Hepatitis C Virus Infections?. Front. Immunol..

[74] Weiss S., Holtfreter S., Meyer T.C., Schmiedeke F., Cammann C., Dörr M., Felix S.B., Grabe H.J., Homuth G., Kohler C. (2023). Toxin Exposure and HLA Alleles Determine Serum Antibody Binding to Toxic Shock Syndrome Toxin 1 (TSST-1) of Staphylococcus Aureus. Front. Immunol..

[75] DeLorenze G.N., Nelson C.L., Scott W.K., Allen A.S., Ray G.T., Tsai A.-L., Charles P., Quesenberry J., Vance G., Fowler J. (2015). Polymorphisms in HLA Class II Genes Are Associated with Susceptibility to Staphylococcus Aureus Infection in a White Population. J. Infect. Dis..

[76] Cyr D.D., Allen A.S., Du G.-J., Ruffin F., Adams C., Thaden J.T., Maskarinec S.A., Souli M., Guo S., Dykxhoorn D.M. (2017). Evaluating Genetic Susceptibility to Staphylococcus Aureus Bacteremia in African Americans Using Admixture Mapping. Genes Immun..

[77] Jaén M., Martín-Regalado Á., Bartolomé R.A., Robles J., Casal J.I. (2022). Interleukin 13 Receptor Alpha 2 (IL13Rα2): Expression, Signaling Pathways and Therapeutic Applications in Cancer. Biochim. Biophys. Acta (BBA) Rev. Cancer.

[78] Washington A., Varki N., Valderrama J.A., Nizet V., Bui J.D. (2020). Evaluation of Interleukin-17D in Host Immunity to Group A Streptococcus Infection. J. Immunol..

[79] Guo S., Zhang J., Li H., Cheng C.-K., Zhang J. (2024). Genetic and Modifiable Risk Factors for Postoperative Complications of Total Joint Arthroplasty: A Genome-Wide Association and Mendelian Randomization Study. Bioengineering.

[80] Chen P.-Y., Wen S.-H. (2024). Integrating Genome-Wide Polygenic Risk Scores with Nongenetic Models to Predict Surgical Site Infection After Total Knee Arthroplasty Using United Kingdom Biobank Data. J. Arthroplast..

